# Creation of an Online Platform for Identification of Microorganisms: Peak Picking or Full-Spectrum Analysis

**DOI:** 10.3389/fmicb.2020.609033

**Published:** 2020-12-18

**Authors:** Konstantin V. Starostin, Evgeny A. Demidov, Nikita I. Ershov, Alla V. Bryanskaya, Vadim M. Efimov, Valeriya N. Shlyakhtun, Sergey E. Peltek

**Affiliations:** ^1^Laboratory of Molecular Biotechnologies of Federal Research Center Institute of Cytology and Genetics of The Siberian Branch of the Russian Academy of Sciences, Novosibirsk, Russia; ^2^Kurchatov Genomics Center of Federal Research Center Institute of Cytology and Genetics of the Siberian Branch of the Russian Academy of Sciences, Novosibirsk, Russia; ^3^Department of Cytology and Genetics, Novosibirsk State University, Novosibirsk, Russia

**Keywords:** biotyping, microorganisms identification, MALDI—TOF, MS data processing, Geometric approach

## Abstract

Identification of microorganisms by MALDI-TOF mass spectrometry is a very efficient method with high throughput, speed, and accuracy. However, it is significantly limited by the absence of a universal database of reference mass spectra. This problem can be solved by creating an Internet platform for open databases of protein spectra of microorganisms. Choosing the optimal mathematical apparatus is the pivotal issue for this task. In our previous study we proposed the geometric approach for processing mass spectrometry data, which represented a mass spectrum as a vector in a multidimensional Euclidean space. This algorithm was implemented in a Jacob4 stand-alone package. We demonstrated its efficiency in delimiting two closely related species of the *Bacillus pumilus* group. In this study, the geometric approach was realized as R scripts which allowed us to design a Web-based application. We also studied the possibility of using full spectra analysis (FSA) without calculating mass peaks (PPA), which is the logical development of the method. We used 74 microbial strains from the collections of ICiG SB RAS, UNIQEM, IEGM, KMM, and VGM as the models. We demonstrated that the algorithms based on peak-picking and analysis of complete data have accuracy no less than that of Biotyper 3.1 software. We proposed a method for calculating cut-off thresholds based on averaged intraspecific distances. The resulting database, raw data, and the set of R scripts are available online at https://icg-test.mydisk.nsc.ru/s/qj6cfZg57g6qwzN.

## Introduction

Due to the advent of the matrix-assisted laser desorption ionization time of flight mass-spectrometry (MALDI TOF MS) and because microorganism identification by means of reference mass spectra libraries has become possible, it is now necessary to choose or develop a new mathematical algorithm for the analysis of mass spectrometry data. Initially, a wide range of mathematical approaches was available for a comparison of mass spectrometry data; these approaches have been tested and optimized on applications related to the identification of organic compounds by gas chromatography with mass spectrometry (Crawford and Morrison, 1968; Grotch, 1970, 1971; Knock et al., 1970; Hertz et al., 1971; Costello et al., 1974; Mathews and Morrison, 1974; McLafferty et al., 1974; Rasmussen and Isenhour, 1979; Stauffer et al., 1985; Stein and Scott, 1994). As a measure for similarity/dissimilarity between spectra, different approaches have been proposed: a sum of difference modules in peak intensities or squares of differences that ended up in one channel (a fixed region of a spectrum) (Crawford and Morrison, 1968; Knock et al., 1970; Grotch, 1971), a ratio of peak intensities in one channel (Hertz et al., 1971), dot-product (Stein and Scott, 1994), Euclidean distance (Rasmussen and Isenhour, 1979), probability-based matching (McLafferty et al., 1974; Stauffer et al., 1985), and more complicated procedures, for example, “divergence” (Farbman et al., 1973). As for biotyping of microorganisms, two main approaches have been assessed: (1) the coefficient of correlation for FSA and (2) peak table-based methods. Coefficients of correlation are a math algorithm for a comparison of two analytical signals presented as functions; this method can also be applied to spectrometry data and to other areas (Horlick and Hieftje, 1978; Ng and Horlick, 1981). In one of the early studies on the identification of microorganisms by MALDI TOF MS, Arnold R.J. and Reilly J.P. used the composite correlation index for a comparison of *E.coli* strains (Arnold and Reilly, 1998). The software developed by them split a spectrum into regions at a given interval, and for each interval, calculated the values of cross-correlation and auto-correlation. The resulting indicator was a mathematical product of the values for all the regions. Their findings showed that such a mathematical approach allows them to successfully discriminate *E.coli* strains. Another example is the work of Dickinson D.N. et al., where they demonstrated that it is possible to discriminate the spores of different species in the genus *Bacillus* (Dickinson et al., 2004a). The correlation coefficient of spectra was calculated point to point. In the next study by the same authors (Dickinson et al., 2004b), aimed at comparing the various methods for identification of microorganisms, it was demonstrated that the newly developed method is not worse than 16S rRNA and gyrB sequence analysis or DNA–DNA hybridization. The approach based on the comparison of full spectra is interesting because it allows researchers to use maximal information contained in the spectra. This strategy may be especially useful for the analysis of spectra with low resolution, which are often MALDI mass spectra of protein profiles of microorganisms. On the other hand, this method is sensitive to noise and to impurities that can be present in a biological sample. Peak table based methods have gained the most popularity and involve compiling a reference fingerprint containing the most significant biomarkers. To compile such a fingerprint, Jarman et al. (1999) have proposed to use a table where each biomarker corresponds to the data based on a series of replicates, including (1) averaged m/z; (2) standard deviation for averaged m/z, (3) averaged relative intensity, (4) standard deviation of intensity, and (5) peak frequency. The pattern-matching algorithm proposed in a subsequent study matches a peak in a test sample with a reference one if its m/z falls into a certain range of reference peak m/z, as determined by the standard deviation for the averaged m/z of the peak (Jarman et al., 2000). By testing for each peak in the analyzed sample, i.e., testing whether it corresponds to the peaks in the reference fingerprint, it is possible to calculate the probability of correspondence of the test fingerprint to the reference fingerprint. Further developments in this approach have led to the creation of the BioTyper software (Sauer et al., 2008), one of the most widespread commercial application. Peak table based methods allow researchers to extract from spectra only the most reliable and significant information but cause the loss of the other information contained in the spectra.

The effectiveness and accuracy of identification of microorganisms directly depend on the availability of a representative database. Existing commercial products are implemented as stand-alone software packages, require regular paid updates, and are tied to a certain type of equipment. Deposition of reference mass spectra into the database is performed by the software developer, whereas in-house databases of the users are hardly accessible to the rest of the user community. Furthermore, commercial databases employ incompatible formats of data storage and methods for spectra processing. The nonprofit sector is also characterized by stand-alone software packages, such as mass-up (López-Fernández et al., 2015) empowered by several R-packages including MALDIquant (Gibb and Strimmer, 2012) and ms-alone, based on R package multiMS-toolbox (Cejnar et al., 2018). Despite supporting the universal formats mzML, mzXML, csv, and others, these software packages do not include an open publicly available database and employ their own custom-designed methods, thereby precluding worldwide integration of mass spectrometry data via the Internet.

To create a publicly available online platform, there should be a single common storage medium accessible for users of mass spectrometers from different vendors as well as a mathematical algorithm that enables processing, classification, and comparison of spectra. To solve this problem, such a Web service should work with the original raw data, meanwhile this calculation burden will be carried by the server part of the project. This arrangement rules out the error that can arise when different methods and parameters are utilized for data processing. This arrangement will also enable investigators to preserve the original information fully contained in the spectra. Most software packages for the work with mass spectra have an option for export of all the data as a txt/csv file, thus helping to address the question of uniformity of the data storage format.

The key task when an online platform is being created is the choice of a data-processing method and of its parameters ensuring the highest accuracy of identification, no inferior to that of commercial products. In a previous work involving the geometric approach, we successfully discriminated two closely related species: *B. pumilus* and *B. altitudinis*, which share >98% homology judging by the 16s rRNA gene sequence. Lists of mass peaks obtained by means of the mMass software were processed via the procedures implemented in the JACOBI package (*jacobi4.ru*). These procedures include the subroutine transforming a list of mass peaks into a multidimensional vector and the functionality for working with vectors in Euclidean space.

In this work, we compare the features of several algorithms for data processing that are based on the geometric approach and incorporate either peak picking analysis (PPA) or full-spectrum analysis (FSA). The methods of mass spectra analysis that involve peak picking identify a set of mass peaks in a spectrum, and the resultant set of peaks serves as a fingerprint for identifying an organism. In the geometric approach to the analysis, the obtained peak list is converted into a multidimensional vector, which is equivalent to describing a spectrum curve at fixed intervals on the m/z axis. Therefore, it makes sense to study the feasibility of comparing spectra by means of full data excluding the peak-picking procedure. This approach allows a researcher to avoid the peak-picking procedure and does not cause a data loss, while still being sensitive to noise. Another problem is a greater (than that for peak picking) volume of stored information and a greater amount of calculations. Nonetheless, the modern advances in computational technologies to some extent have reduced this problem (Cejnar et al., 2018). As a model for analysis, here we chose 74 strains that mostly represent different species of the genus *Bacillus*. We investigated the influence of various factors on the accuracy of identification, e.g., such factors as normalization procedures, intensity transform, parameters of spectra processing, and the cutoff of relative intensity during the peak search. We also proposed a solution to the problem of the development of the method for calculating the cutoff criterion for the results of identification at the species level.

## Materials and Methods

### Description of the Group of Microbial Strains and Microbiological Procedures

Seventy-four strains from the collections of ICG SB RAS (Collection of biotechnological microorganisms as a source of novel promising objects for biotechnology and bioengineering of Institute of Cytology and Genetics, Siberian Branch of Russian Academy of Sciences), UNIQEM (Collection of Unique and Extremophilic Microorganisms of various physiological groups for biotechnological purposes of the Research Center of Biotechnology RAS), IEGM (Collection of Institute of Ecology and Genetics of Microorganisms, Ural Branch of Russian Academy of Sciences), KMM (Collection of Marine Microorganisms, Pacific Institute of Bioorganic Chemistry, Far Eastern Branch of Russian Academy of Sciences), and VKM (All-Russian Collection of Microorganisms, G.K.Skryabin Institute of Biochemistry and Physiology of Microorganisms, of Russian Academy of Sciences) were used. This set of strains represents the following species: the *Bacillus cereus* group (14 strains), *Bacillus pumilus* (18 strains), *Bacillus altitudinis* (nine strains), *Bacillus aryabhattai* (one strain), *Bacillus berkeleyi* (one strain), *Bacillus subtilis* (one strain), *Bacillus simplex* (six strains), *Bacillus megaterium* (six strains), *Bacillus coagulans* (one strain), *Bacillus chungangensis* (one strain), *Bacillus atrophaeus* (two strains), *Bacillus clausii* (one strain), *Bacillus flexus* (two strains), *Bacillus licheniformis* (eight strains), *Anoxybacillus gonensis* (one strain), *Geobacillus subterraneus* (one strain), *Geobacillus jurassicus* (one strain), and *Escherichia coli* (one strain). Detailed information on the strains and their cultivation conditions can be found in the Supplement.

The studied strains were isolated from geographically and environmentally diverse locations, from Kamchatka hot springs to saline lakes of Southern Siberia. We isolated microbial strains from high-temperature petroleum reservoirs, freshwater and saline water bodies, thermal springs, tailings dams, rhizospheres of higher plants, etc. The samples were taken from both pristine and polluted locations. Strains were cultivated on diverse media: LB, PCA, malt agar, potato agar, at 28–60°C.

The isolated strains include those found in air, soil and water; thermophilic and mesophilic; acidophilic, neutrophilic, and alkaliphylic; halophylic and freshwater.

Despite being phylogenetically closely related, the studied strains obviously possess a very broad diversity of phenotypic characters.

### Mass-Spectrometric Analysis

For this analysis, a full microbiological loop of a given culture was transferred into a 1.5 ml Eppendorf tube and resuspended in 300 μl of deionized water. For inactivation of bacterial cells, 900 μl of ethanol was added with thorough mixing. The cells were collected by centrifugation for 2 min at 15,600 × g, the supernatant was discarded, and the pellet was dried for 5 min in a vacuum concentrator (Eppendorf Concentrator Plus). The cell walls were disrupted by the addition of 50 μl of 70% formic acid. For extraction of proteins, to the resulting mixture, 50 μl of acetonitrile was added followed by thorough mixing. The obtained mixture was centrifuged for 2 min at 15,600 × g, and the supernatant was transferred into a fresh microfuge tube for subsequent mass-spectrometric analysis.

For this analysis, 1 μl of the protein extract was applied to a stainless-steel plate and allowed to dry at room temperature. After that, the sample was overlaid with 1 μl of the matrix [6 mg/ml α-cyano-4-hydroxy-cinnamic acid in an acetonitrile/water/trifluoroacetic acid mixture (50:47.5:2.5, v/v/v)].

The spectra were recorded on an Ultraflex III MALDI time-of-flight TOF/TOF mass spectrometer (Bruker Daltonics). They were acquired in linear positive mode at laser frequency 100 Hz in a mass range of 2,000–20,000 Da. Voltage on the accelerating electrode was 25 kV, voltage IS2 23.45 kV, and voltage on the lens 6 kV, without delays of extraction.

External calibration was conducted by means of precise mass values of known proteins of *E. coli*: RL36,4365.3 Da;RS22,5096.8 Da;RL34,5381.4 Da;RL32,6315.0 Da;RL29,7274.5 Da; and RS19,10300.1 Da.

To build the reference database, 12 colonies of each strain were chosen randomly. For creation of the test database, three colonies of each strain were collected. For each colony, three spectra were recorded by summing 500 laser impulses (5 × 100 impulses from different positions of the target cell). The mass spectra were inspected visually.

### Processing of the Mass Spectra

**Figure 1:**
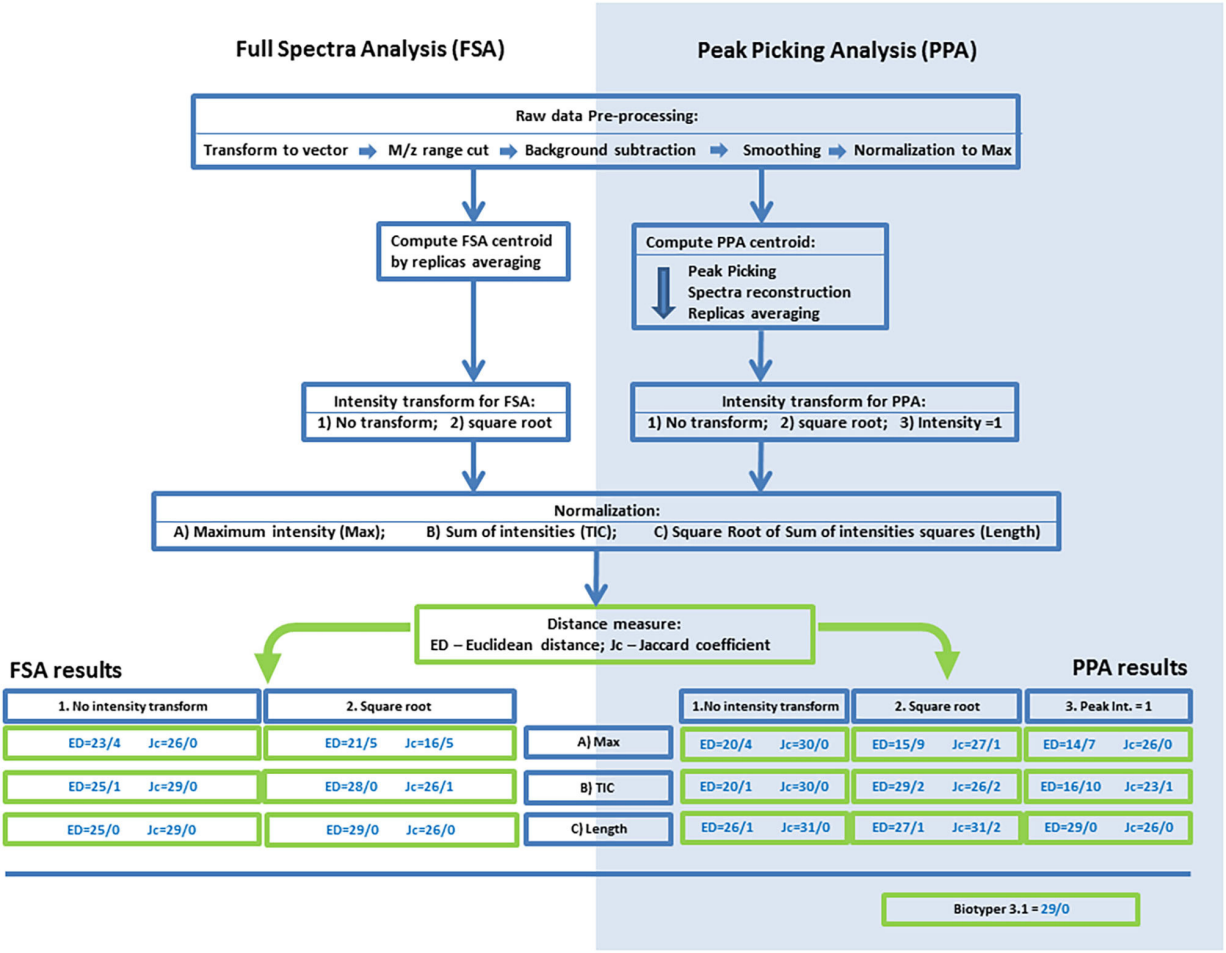
An outline of the processing procedures and analyses of mass spectrometry data for the algorithms based on full-spectrum analysis (FSA) or peak-picking analysis (PPA). Results of the FMS/FMM test are presented for various combinations of normalization, centroid intensity transform, and distances. The following codes and abbreviations were used to denote algorithms and procedures: Max, normalization to maximal intensity; TIC, normalization to the sum of intensities; Length, normalization to the root of the sum of squares of intensities; NT, no intensity transform; SRT, square root transform; PP1, setting the intensities of peaks to 1.0. The full name of an algorithm is composed of the analysis method (FSA or PPA), code of intensity transform, code of normalization, and the metric (ED or Jc).

RAW spectra were exported using the mMass software, as a plain-text table (m/z;I). For the reference dataset, all replicates were exported independently. For the test dataset, averaged data were exported from mMass to quicken the R script. The data were processed with an R script in the R3.4.3 software. Publicly available libraries MALDIquant, baseline were used in this work. An outline of the processing of mass spectrometry data is presented in Figure 1.

#### Raw-Data Processing

Exported mass spectra were converted into a data array (m/z_i_; I_i_) with a fixed step of one Da along the *m*/*z* axis. The resulting vector was truncated to a given range on them/*z* axis. For baseline extraction, iterative restricted least squares (IRLS) (Jorgensen, 2006; Garvey et al., 2015), Friedman's super smoother (Friedman, 1984) (FSS), and sensitive nonlinear iterative peak-clipping (SNIP) (Morháč and Matoušek, 2008) were employed. Smoothing was conducted via dynamic rolling mean and undecimated discrete wavelet transform (UDWT) (Percival and Walden, 2000) procedures. The resulting vector was normalized to maximal intensity.

#### Generation of a Centroid

For PPA, we chose the windowed local maximum procedure for peak searching with the following parameters: intensity threshold 1, 5, and 10%, half-window size = 1, and signal-to-noise-ratio = 3. Reconstruction of peak lists in the form of a vector was carried out using a polynomial function as described elsewhere (Starostin et al., 2015). The centroids for PPA and FSA were calculated as an arithmetic mean of the vectors constructed for each strain.

#### Intensity Transform

No intensity transform (NT), square root transform (SRT), or a reduction of all peaks' intensities to 1.0 (PP1) we performed in the case of PPA.

#### Filtering of Low-Intensity Signals

To get rid of low-intensity noise in the case of FSA algorithms, intensities below a certain threshold were set to zero.

#### Normalization of Centroids

To maximal intensity (here after: Max), to the sum of intensities (TIC), onto the square root of the sum of squares of intensities (Length) was carried out.

### A Comparison of Centroids

Similarity/dissimilarity between centroids were measured by Euclidean distance (ED) and Jaccard (Jaccard, 1901; Marczewski and Steinhaus, 1958; Gower, 1985) (Jc) coefficient. All the comparison results were sorted in the order of either increasing EDs or decreasing Jc coefficients. Either the lowest ED or the highest Jc coefficient was assumed to denote the best match between the test sample and a sample in the reference database.

### The FMS/FMM Test

The effectiveness of PPA-based and FSA-based algorithms is assessed via the lowest number of false matches (FMM), when the best match occurs between the centroids of different species, and via the greatest number of matches at the strain level (FMS), when the test and reference centroids separated by the shortest distance belong to the same strain.

### Computation of the Cutoff as a Criterion

To this end, we utilized four subsets of strains belonging to species *Bacillus simplex, Bacillus licheniformis, Bacillus megaterium*/*aryabhattai*, and to the *Bacillus cereus* group. For each subset, we computed an intragroup average distance between the centroids of strains. As a cutoff, we chose the averaged-by-species value of intragroup distances.

### Identification of Microorganisms by Means of the Cutoff

**Figure 2:**
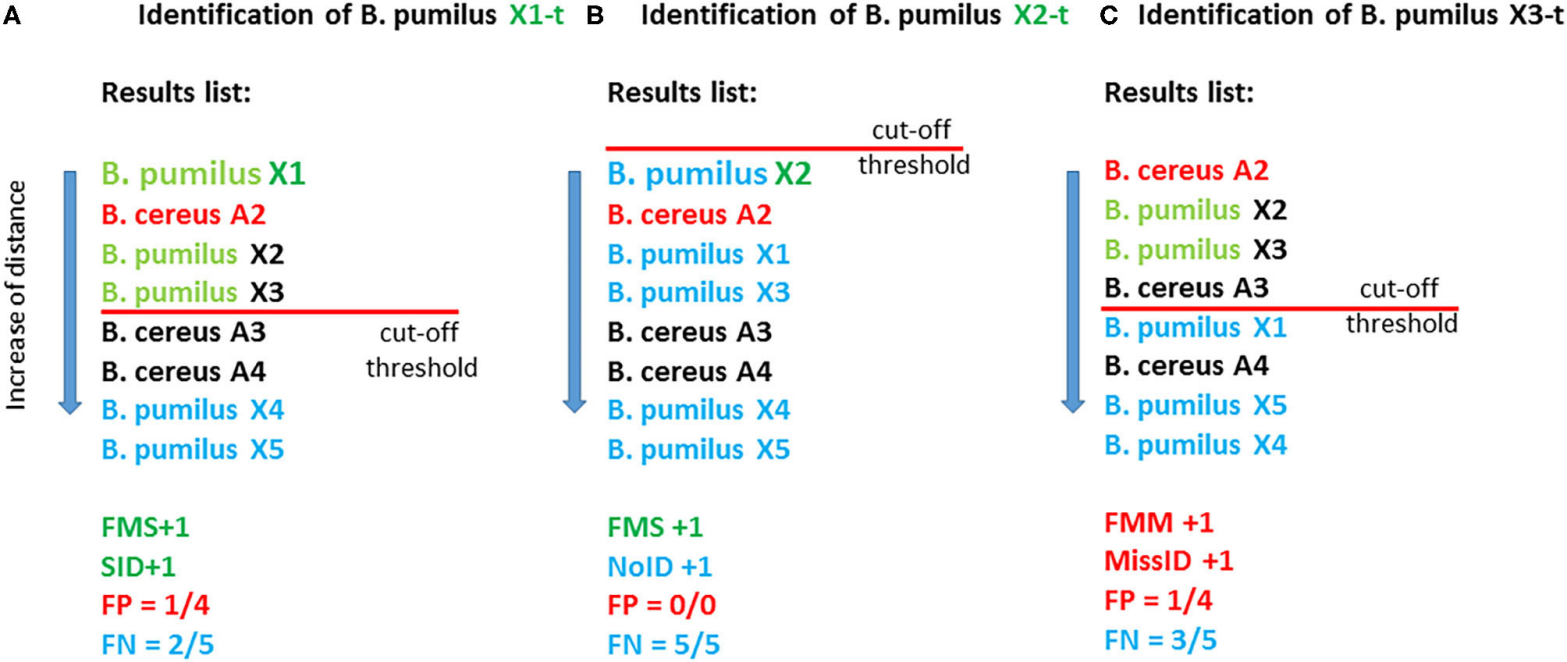
Illustrated computation of the parameters of effectiveness of the methods with the use of a cutoff. In this fictional example, the database contains eight reference entries (eight samples): five *B. pumilus* entries and three *B. cereus* entries. As test samples, X1-t, X2-t, and X3-t are used which correspond to the reference samples of *B. pumilus* strains X1, X2, and X3. **(A)** The best match for X1-t is X1; therefore, FMS is +1, and the distance for this match is less than the cutoff, and consequently SID is +1. In total, this criterion (cutoff) is met by four matches, and among them, three matches correspond to “own” species *B. pumilus*, and one match corresponds to “another” species: *B. cereus* (reference sample *B. cereus* A2). Therefore, parameter FP equals ¼, i.e., 25%. Two matches out of five possible for the species *B. pumilus* (X4 and X5) did not pass the cutoff; therefore, FN = 2/5 = 40%. **(B)** The best match for X2-t is X2; therefore, FMS +1. Nonetheless, in this case, not a single match passed the cutoff, and consequently NoID +1, FP = 0%, FN = 5/5 = 100%. **(C)** The best match for *B. pumilus* X3-t is *B. cereus* A2; therefore, FMM+1. This match passed the cutoff, and therefore MissID +1.

Identification of the test samples was conducted in the first sample in a list if the distance to the reference sample was less than the cutoff. StrainID means the best match conforming to the cutoff on the condition that both the test sample and reference sample belong to one strain. If the first sample passes the cutoff and belongs to a wrong species, then it is regarded as falsely identified: Miss ID. The average percentage of “false” samples relative to the total number of samples that passes the cutoff is defined as the percentage of false positive results: FP. If the distance to the best match is greater than the cutoff, then the sample is regarded as unidentified: NoID. The average percentage of samples belonging to the species of the tested sample that do not pass the cutoff is considered a false negative result: FN. The computation of the above criteria is depicted in Figure 2.

### Cross-Validation

To perform cross-validation, the test samples were identified one after another using the reference database. From the results of the comparison with the reference database, we excluded the case when both the test sample and reference sample belonged to one strain. Identification was conducted via the FMS/FMM test and the cutoff.

## Results

### Parameter Optimization of the Procedures for Mass Spectra Processing

The procedures of subtraction of baseline (IRLS, FSS and SNIP) were evaluated. Result quality was inspected visually. Optimal results were obtained using SNIP with the window parameter set to 10. Parameter optimization of the smoothing procedure and determination of the optimal range of m/z were carried out by the FMS/FMM test. The best results were obtained by means of dynamic rolling mean with the starting and final window set to 1 and 3, respectively, and the m/z range of 3–15 kDa.

For the peak-picking procedure, the filter parameter was optimized by minimal relative intensity. The study of this parameter's role helped to assess the contribution of low-intensity peaks to the species specificity of spectra. Threshold values 1, 5, and 10% were assessed. The results of identification of a set of test samples for these parameters were as follows:

1%. ED (FMS/FMM): 20/4, Jc (FMS/FMM): 29/0

5%. ED (FMS/FMM): 21/5, Jc (FMS/FMM): 26/0

10%. ED (FMS/FMM): 19/6, Jc (FMS/FMM): 28/2

When the threshold exceeded 1%, the accuracy of identification diminished, pointing to the importance of low-intensity peaks for the species specificity of a mass spectrum. Judging by the results, the optimal threshold was 1%.

For FSA-based algorithms, we applied the procedure of filtration of low-intensity noise. Thresholds 0, 0.5, 1, and 5% were evaluated. The results of identification of a set of test samples for these parameters were as follows:

0%. ED (FMS/FMM): 22/4, Jc (FMS/FMM): 22/2

0.5%. ED (FMS/FMM): 23/4, Jc (FMS/FMM): 26/0

1%. ED (FMS/FMM): 22/4, Jc (FMS/FMM): 26/0

5%. ED (FMS/FMM): 20/6, Jc (FMS/FMM): 24/1

Thus, the optimal threshold was 0.5–1.0%, and in subsequent analyses, 0.5% was chosen for the filtration of low-intensity noise.

### A Comparison of the Effectiveness of PPA-and FSA-Based Algorithms by the FMS/FMM Test

The overall processing algorithm consisted of sequential operations: preprocessing, computation of a centroid, normalization, intensity transformation, and calculation of distances. On the basis of the several types of normalization and intensity transform and the two methods for calculation of the distance (ED and Jc), we obtained 30 algorithms for the processing and comparison of mass spectrometry data. The initial evaluation of these algorithms and the search for the most effective ones were performed by the FMS/FMM test. The results are shown in Figure 1.

### FSA

In FSA, the best approach is Length normalization, and the worst is Max normalization, regardless of the metric used or the method for intensity transform. With SRT, in the case of Max normalization, the worst results were obtained: 21/5 for ED and 16/5 for Jc. With Tic or Length normalization, SRT was found to be more effective for ED, and no transform was more effective for Jc. In case of ED, the best algorithm was Length-SRT, whereas for Jc, the best algorithm was Length-NT; both yielded the 29/0 result. These algorithms were selected for subsequent analyses.

### PPA

The results were noticeably different between ED and Jc. In case of ED, the highest effectiveness was achieved with Length normalization, and the lowest with Max normalization. The reduction of peak intensities to 1.0 notably worsened accuracy for Max normalization and Tic normalization but yielded the best result with Length normalization: 29/0. The square root of intensity gave good results with TIC or Length normalization, and the worst result with Max normalization: 15/9. It can be concluded that for this metric, Length normalization is the best choice, and in the case of the intensity transform, all three types are worthwhile. For Jc, the variance of the results was substantially lower than that with ED: from 26/2 to 31/0. The transform via the square root worsened the results. Within one type of transform of intensities, all three types of normalization yielded similar results. For subsequent analyses, methods Length-PP1 and Length-NT were chosen as the best options for the metrics ED and Jc, respectively.

As an external control, the FMS/FMM test was performed on the results of identification in Biotyper 3 software. The obtained value was 29/0, which is worse than that for the most effective methods of PPA and comparable with the results of FSA-based methods.

### Identification With the Use of the Cutoff

For each of the four selected algorithms, the cutoff was computed. To evaluate selectivity, the criteria were first tested on the reference database. In this case, the reference database entries were identified by means of the same database. FP and FN are presented in Table 1 for each algorithm in a row with its name. FN values did not show substantial variance and were within the range of 37–48%, owing to the method of criterion calculation. FP values did not exceed 10% for the methods based on peak picking, whereas in the case of PPA-Length-NT-Jc, FP was only 1.3%, indicating high selectivity of these methods. For methods FSA-Length-SRT-ED and FSA-Length-NT-Jc, FP was 10.8 and 18.2%, respectively.

**Table 1:** Results of identification of the test set of samples by means of the calculated cutoffs and the reference collection of samples.

	**FP, %**	**FN, %**	**FMS**	**SID**	**FMM**	**MissID**	**NoID**
**PPA-Length-PP1-ED (FP/FN: 10/48%)**							
Test 1	6.9	61.5	29	27	0	0	5
Test 2	11.1	59.5	29	26	4	4	6
Crossvalid.	12.6	71.3			6	4	13
**PPA-Length-NT-Jc (FP/FN: 1.3/47.1%)**							
Test 1	2.7	52.5	31	30	0	0	2
Test 2	3.6	52.1	31	30	0	0	4
Crossvalid.	2.7	62.1			4	1	10
**FSA-Length-SRT-ED (FP/FN: 10.8/43.8%)**							
Test 1	0.9	76.4	29	18	0	0	25
Test 2	2.2	72.3	29	18	1	1	28
Crossvalid.	1.9	76.5			3	1	29
**FSA-Length-NT-Jc (FP/FN: 18.2/44.3%)**							
Test 1	3.4	63	29	27	0	0	9
Test 2	4.7	62	29	22	1	1	16
Crossvalid.	4.3	67			4	1	16
**Biotyper**							
Test 1	1.3	46	29	27	0	0	3
Test 2	3	41.5	29	27	0	0	3
Crossvalid.	2.1	51.3			4	0	11

*(1) FP (false positives, the percentage of reference samples belonging to another species that pass the cutoff in the results on the identification of the test set); (2) FN (false negatives, the percentage of reference strains from the same species that do not pass the cutoff in the results on the identification of the test set); (3) Strain ID (the best match that passed the cutoff at the strain level); (4) Miss ID (the best match that passes the cutoff but belongs to a different species); (5) NoID (no matches that passed the cutoff). The FP/FN values calculated using the reference database are presented in a row called “algorithm.” Test 1, B. pumilus and B. altitudinis were regarded as one group. Test 2, B. pumilus and B. altitudiniswere considered different groups. Crossvalid., the results of cross-validation conducted with sequential exclusion of a sample identical to the test sample from the reference collection*.

Next, we carried out the identification of a set of test samples with the use of the cutoff (Table 1). Figure 2 details the algorithms for calculation of the parameters of methods' effectiveness. Some cases are reviewed separately: when closely related species *B. pumilus* and *B. altitudinis* were regarded as a single group (test 1) or two different groups (test 2). The cutoff was computed separately in each case.

Algorithm PPA-Length-NT-Jc yielded the best results: 30 instances of identification at the strain level in the absence of instances of incorrect identification. This performance is better than that of Biotyper 3. FSA-Length-SRT-ED gave the worst result: 18 and 0, respectively. A comparison of Test1 and Test2 revealed that the majority of methods could successfully discriminate closely related strains of *B. pumilus* and *B. altitudinis*. A noticeable increase in the number of instances of incorrect identification takes place only in the case of algorithm PPA-Length-PP1-ED. For our PPA-based algorithms, FP and FN values were comparable between reference database tests (test1 and test2), thus indicating good reproducibility of the biological replicates and good selectivity of the algorithms, especially in the case of PPA-Length-NT-Jc. When FSA-based algorithms were tested, parameter FN was substantially greater. This finding means greater discrimination between biological replicates, which are represented by test and reference sets of samples. As a consequence, the cutoff calculated via the reference set of samples turned out to be too restrictive, thereby leading to both low FP and high NoID.

### Cross-Validation

For this purpose, a sample corresponding to a test sample was excluded from the reference database. Because the species *E. coli, G. subterraneus, G. jurassicus, B. berkeleyi, B. coagulans, B. subtilis, A. gonensis, B. chungangensis*, and *B. clausii*are each represented by a single sample, they were excluded from the list of test samples. In the same way, we excluded *B. atrophaeus* and *B. flexus*, which were represented by two samples each. The results are listed in Table 1. The proposed method of cross-validation virtually eliminates the possibility of identifying a sample by its own biological replicate (FMS and SID), thereby notably increasing the number of mismatches in terms of the comparison with the first sample in a list (FMM). At the same time, MissID is not higher because mismatches are filtered out by the cutoff. All the algorithms except PPA-Length-PP1-ED were not inferior to Biotyper 3 according to the results of cross-validation.

## Discussion

The aims of this work were to compare two approaches to the analysis of mass spectrometry data and to develop a convenient and reliable cutoff for the identification of unknown samples. As an experimental model, we used the strains mostly belonging to the genus *Bacillus* (we also included some representatives of genera *Geobacillus, Anoxybacillus*, and *Escherichia)*. The largest subset including 14 strains [B504, B13, KUskv2(1), B82, 44(7)il, O43, 46(10)il, 41(4)il, UDO1, 41(7)il, B370, KU82(2), 666, and 664] belongs to the *B. cereus* group which contains species *B. cereus, B. thuringiensis, B. toyonensis, B. mycoides*, and *B. weihenstephanensis*. These species share very high genetic similarity and are difficult to discriminate by 16S rRNA gene sequencing (Rasko et al., 2005). For this reason, in this study, we did not discriminate these strains at the species level. Besides, we regarded the strains belonging to *B. megaterium* and *B. aryabhattai* as one group.

A solution to the problem of finding an algorithm for the processing and analysis of mass spectrometry data is relevant and important for a publicly available online platform designed for working with an open database. The algorithms currently in use are based on a peak-picking procedure. This approach allows for the removal of noise and of matrix effects and is most productive in terms of calculation speed. Nevertheless, it causes an unavoidable loss of data for the following reasons:

1. Poorly resolved peaks may be registered as one peak or only the more intensive peak will be taken into account.
2. A restrictive threshold may exclude relevant low-intensity peaks.
3. Restrictive statistical criteria can discard peaks of low frequency.

In the work with clinical strains, commercial platforms employ standardized conditions of growth, sample preparation from cultures, equipment, and software. Under such conditions, the drawbacks of peak picking are minimized by the high reproducibility of mass spectra. By contrast, in the work with natural strains, which feature substantial diversity of growth conditions, it becomes impossible to find optimal conditions for growth and sample preparation during high-throughput screening. This state of affairs will decrease reproducibility. “Nonstandard” results will be caused by the isolation of cultures on specific and sometimes toxic substrates or under specific conditions for growth: e.g., extreme pH levels, temperatures, and ionic strength. Another downside of this problem is the reproducibility of results on various brands and models of mass spectrometers.

A possible solution to this problem is algorithms based on FSA, which minimize the data loss. This approach mostly resolves the issue of poorly resolved peaks. During the calculation of a centroid, peaks of low frequency will be taken into account to the extent that they contribute to the centroid. The trouble with low-intensity peaks still depends on the effectiveness of the algorithms for noise filtration and is equally inherent in both FSA and PPA.

Aside from the comparison of two principal approaches, we studied the influence of various procedures for the processing of mass spectrometry data on identification accuracy. As a metric for the comparison, ED and the Jc coefficient were employed, which are coefficients of correlation that can be converted to the1-Jc metric.

Our initial analysis based on the comparison of indicators FMS/FMM revealed an advantage of Jc as a metric for data comparison in case of PPA-based algorithms. In the case of FSA-based algorithms, this metric similarly either had an advantage over (or was comparable to) ED usually. The geometric approach in the proposed version of the method constructs a full spectrum from peak lists or directly transforms raw mass spectrometry data onto a coordinate plane as a multidimensional vector. Consequently, correlation analysis is an effective method for dissecting such data. Conversion into the 1-Jc metric enables investigators to effectively apply “geometric” methods of cluster analysis.

Depending on the metric in question, Jc or ED, different types of normalization had different effects on the results of identification of the strains. In the case of ED, the accuracy of identification decreased in the order Length, TIC, and Max. In the case of 1-Jc, the impact of normalization was insignificant.

The square root transform allows increasing the relative contribution of low-intensity peaks. This transform appreciably improved the results with Length or TIC normalization, suggesting that these peaks are important for accurate identification.

Setting the intensities of peaks to 1.0 before their transformation into a multidimensional vector allows researchers to remove a factor called relative intensity from consideration and thus to assess its usefulness for species specificity of the spectra. With normalization to maximal intensity or the sum of intensities, this transform caused a notable decrease in the accuracy of algorithms. By contrast, with normalization to Length, the accuracy was similar or better than that of known effective methods. This finding points to the predominant role of the m/z values of mass peaks in the species specificity of mass spectra; a simple list of mass peaks is sufficient for satisfactory identification at the species level. Nonetheless, relative intensity of the peaks remains a relevant parameter making the method more reliable. If we take a look at a more detailed investigation of the method, we will notice that the PPA-Length-1-ED method miserably fails at the identification of closely related species: Test 2 and cross-validation with the exclusion of a biological replicate. In our previous work, we learned that the differences between species *B. pumilus* and *B. altitudinis* are mostly explained by several high-intensity peaks. With the removal of information about relative intensity, this distinction for the most part disappears.

**Figure 3:**
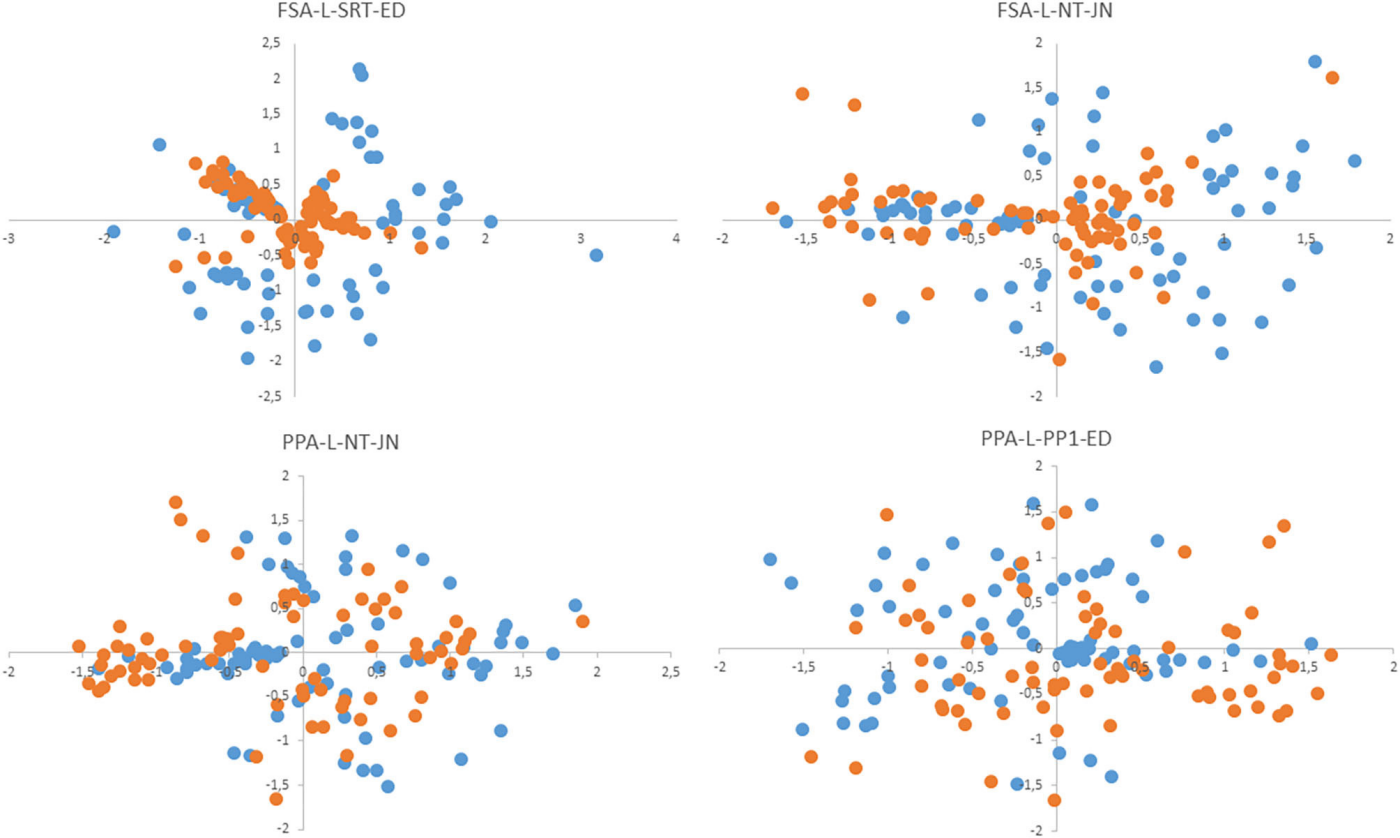
The nonmetric multidimensional scaling (NMDS) plot shows the positions of centroids of test samples (blue dots) and reference samples (red dots) relative to each other.

The method proposed here for computation of the cutoff derives from the hypothesis that the average distance between centroids of strains belonging to one species will be a characteristic feature. Our comparison of average intraspecies differences for some taxa (the *B. pumilus* group, *B. cereus* group, *B. megaterium, B. licheniformis*, and *B. simplex)* showed that its value is comparable among different species. Therefore, the averaged value was utilized as a characteristic feature indicating how close the tested sample is to the reference sample in order to assign it to the corresponding species. Parameter FP calculated on the basis of the cutoff is the percentage of other species' strains that passed this filter. This parameter characterized the selectivity of an algorithm, that is, how far the “clouds” of different species are located relative to one another in multidimensional space. High FP increases the probability of false identification, especially in sets of samples with low representativeness. Parameter FN is the percentage of strains from the same species that do not pass the cutoff. During the identification of the test set, this parameter indicates the distance between the test set and reference set of samples. In Figure 3, this notion is illustrated as a two-dimensional nonmetric—scaling plot, which simultaneously presents the centroids of reference and test samples. PPA-based algorithms manifested lower parameter FN (as compared to FSA-based ones) and accordingly a high percentage of identified strains.

In brief, implementation of the geometric approach substantially improves analytical characteristics for the identification of microorganisms. This principle is especially important when it is impossible to strictly standardize the methods of cultivation, sample preparation, and acquisition of primary mass spectrometry information, as strictly as in the case of clinical diagnostics. The evaluated algorithms based on either PPA or FSA showed comparable or greater effectiveness than Biotyper 3.1 software did. FSA-based algorithms are somewhat worse than PPA-based ones. We attribute this finding to the sensitivity for low-intensity noise; however, it seems that FSA-based algorithms may have an advantage when reference and test spectra have different resolution. A possible solution to this problem is to find a more effective algorithm for noise filtration. Both approaches may serve as the basis for the creation of an open online platform for microorganism identification. The proposed technique for the computation of the cutoff and the metrics also manifested high accuracy of identification. A special advantage of the proposed method for cutoff calculation is the simplicity of computation, which will help to rapidly adjust its values during the growth of the database.

In the course of this work, a database of reference mass spectra was compiled consisting of 74 strains. The algorithm for the processing and analysis of mass spectrometry data is implemented as an R script and will serve as the basis for the mathematical part of the online platform helping to work with mass spectrometry data. Raw data and the R script are available at https://icg-test.mydisk.nsc.ru/s/qj6cfZg57g6qwzN.

## Data Availability Statement

The original contributions generated for the study are publicly available. This data can be found here: https://icg-test.mydisk.nsc.ru/s/qj6cfZg57g6qwzN.

## Author Contributions

KS, ED, and SP wrote the main manuscript text. KS performed mass-spectrometry analysis, computations, and results analysis. VE, NE, KS, and ED developed the mathematical algorithm. NE developed the R script. AB performed microbiological works. VS performed 16s rRNA sequencing. SP led the project. All authors contributed to the article and approved the submitted version.

## Conflict of Interest

The authors declare that the research was conducted in the absence of any commercial or financial relationships that could be construed as a potential conflict of interest.
