# Supplementary material for: Creation of an Online Platform for Identification of Microorganisms: Peak Picking or Full-Spectrum Analysis
Source: Front Microbiol. 2020 Dec 18;11:609033. doi: 10.3389/fmicb.2020.609033 (PMC7775396; doi:10.3389/fmicb.2020.609033)
Supplement: Supplementary file 1 [file Table_1.docx]

**Supplement**

Description of the strains used in this work. The following media were employed: LB, Luria–Bertani; MPA, meat peptone agar

| **#** | **Species** | **Code** | **Growth conditions** | **GenBank accession** |
| --- | --- | --- | --- | --- |
| UNIQEM | | | |  |
| 1 | *Bacillus cereus* | B-504 | MPA, 30°С | AF290547.1 |
| 2 | *Bacillus pumilus* | Antclone 1 | MPA, 30°С | MN447144 |
| 3 | *Geobacillus subterraneus* | 34Т | PCA, 60°С | AF276306 |
| 4 | *Geobacillus jurassicus* | DS1T | PCA, 60°С | AY312404 |
| IEGM | | | |  |
| 5 | *Bacillus mycoides* | 666 | PCA, 30°С (MPA, LB) | MN447139 |
| 6 | *Bacillus aryabhattai* | 2021 | LB, 30°С (MPA) | MN447140 |
| KMM | | | |  |
| 7 | *Bacillus berkeleyi* | 6244 | MPA, LB, 28°С | NR109459.1 |
| 8 | *Bacillus subtilis* | 6806 | MPA, LB, 28°С | MN447142 |
| 9 | *Bacillus pumilus* | 3884 | MPA, LB, 28°С | MN447141 |
| 10 | *Bacillus simplex* | 9419 | MPA, LB, 28°С | MN447143 |
| VKM | | | |  |
| 11 | *Bacillus megaterium* | B-40 | MALT AGAR, LB, 30°С | MN447133 |
| 12 | *Bacillus megaterium* | B-396 | MALT AGAR, LB, 30°С | MN447137 |
| 13 | *Bacillus megaterium* | B-394 | MALT AGAR, LB, 30°С | MN447136 |
| 14 | *Bacillus megaterium* | B-397 | POTATO AGAR, LB, 30°С | MN447138 |
| 15 | *Bacillus thuringiensis* | B-82 | POTATO AGAR, LB, 30°С | MN447134 |
| 16 | *Bacillus megaterium* | B-512T | POTATO AGAR, LB, 28°С | NR112636.1 |
| 17 | *Bacillus coagulans* | B-497 | MPA, LB, 30°С | NR041523 |
| 18 | *Bacillus thuringiensis* | B-370 | MPA, LB, 30°С | MN447135 |
| 19 | *Bacillus cereus* | B-13 | MPA, LB, 30°С | MN447132 |
| ICG SB RAS | | | |  |
| 20 | *Anoxybacillus gonensis* | Seya | LB, 60°С | KY323316 |
| 21 | *Bacillus chungangensis* | 45(3)il | LB, 37°С | KY323317 |
| 22 | *Bacillus megaterium* | 45(5)il | LB, 37°С | KY323318 |
| 23 | *Bacillus simplex* | 47(5)w | LB, 37°С | KY323319 |
| 24 | *Bacillus simplex* | 41(2)w | LB, 37°С | KY323320 |
| 25 | *Bacillus simplex* | KL20 | LB, 37°С | KY323321 |
| 26 | *Bacillus simplex* | O4 | LB, 37°С | KY323322 |
| 27 | *Bacillus simplex* | O45 | LB, 37°С | KY323323 |
| 28 | *Bacillus atrophaeus* | 47(8)il | LB, 37°С | KY323324 |
| 29 | *Bacillus atrophaeus* | 47(11)il | LB, 37°С | KY323325 |
| 30 | *Bacillus thuringiensis* | 41(7)il | LB, 37°С | KY323326 |
| 31 | *Bacillus cereus* | 44(7)il | LB, 37°С | KY323327 |
| 32 | *Bacillus toyonensis* | 46(10)il | LB, 37°С | KY323328 |
| 33 | *Bacillus thuringiensis* | O43 | LB, 37°С | KY323329 |
| 34 | *Bacillus cereus* | 41(4)il | LB, 37°С | KY323330 |
| 35 | *Bacillus thuringiensis* | KUskv2(1) (K9dt) | MPA, 37°C | KY323331 |
| 36 | *Bacillus thuringiensis* | UDO1 | MPA, 37°C | KY323332 |
| 37 | *Bacillus mycoides* | KU82(2) (K24dt) | MPA, 37°C | KY323333 |
| 38 | *Bacillus clausii* | 13U | MPA, 37°C | KY323334 |
| 39 | *Bacillus flexus* | 4U | MPA, 37°C | KY323335 |
| 40 | *Bacillus flexus* | 42(1)il | MPA, 37°C | KY323336 |
| 41 | *Bacillus licheniformis* | 2U | MPA, 37°C | KY323337 |
| 42 | *Bacillus licheniformis* | 9U | MPA, 37°C | KY323338 |
| 43 | *Bacillus licheniformis* | 15U | MPA, 37°C | KY323339 |
| 44 | *Bacillus licheniformis* | 16U | MPA, 37°C | KY323340 |
| 45 | *Bacillus licheniformis* | KG16(1)2011 (K14dt) | MPA, 37°C | KY323341 |
| 46 | *Bacillus licheniformis* | KU16oc(1)2011 (KH11) | MPA, 37°C | KY323342 |
| 47 | *Bacillus licheniformis* | KU16oc(3)2011 (K5dt) | MPA, 37°C | KY323343 |
| 48 | *Bacillus licheniformis* | KUskv2t1(1) | MPA, 37°C | KY323344 |
| 49 | *Bacillus altitudinis* | Cd3 | MPA, 37°C | KY323345 |
| 50 | *Bacillus pumilus* | О48 | LB, 37°C | KP699776 |
| 51 | *Bacillus pumilus* | О32 | LB, 37°C | KP699772 |
| 52 | *Bacillus pumilus* | O19 | LB, 37°C | KP699775 |
| 53 | *Bacillus pumilus* | O6 | LB, 37°C | KP699774 |
| 54 | *Bacillus pumilus* | 47(6)il | S4, 37°C | KP699765 |
| 55 | *Bacillus pumilus* | 51(3)w | LB, 37°C | KP699766 |
| 56 | *Bacillus pumilus* | O41 | LB, 37°C | KP699767 |
| 57 | *Bacillus pumilus* | O33 | LB, 37°C | KP699768 |
| 58 | *Bacillus pumilus* | 48(1)w | LB, 37°C | KP699778 |
| 59 | *Bacillus pumilus* | 46(5)il | LB, 37°C | KP699764 |
| 60 | *Bacillus pumilus* | 51(1)il | LB, 37°C | KP699773 |
| 61 | *Bacillus pumilus* | 51(5)il | LB, 37°C | KP699777 |
| 62 | *Bacillus pumilus* | 42(6)w | LB, 37°C | KP699769 |
| 63 | *Bacillus pumilus* | 3U | MPA, 37°C | KP699770 |
| 64 | *Bacillus pumilus* | 10U | MPA, 37°C | KP699771 |
| 65 | *Bacillus altitudinis* | КН6 | MPA, 37°C | KP699782 |
| 66 | *Bacillus altitudinis* | KU3-5(2) | MPA, 37°C | KP699787 |
| 67 | *Bacillus altitudinis* | КН2 | MPA, 37°C | KP699786 |
| 68 | *Bacillus altitudinis* | KG16(2) | MPA, 37°C | KP699780 |
| 69 | *Bacillus altitudinis* | К6dt | MPA, 37°C | KP699783 |
| 70 | *Bacillus altitudinis* | КН3 | MPA, 37°C | KP699785 |
| 71 | *Bacillus altitudinis* | KG16(3) | MPA, 37°C | KP699781 |
| 72 | *Bacillus altitudinis* | Cd1 | MPA, 37°C | KP699779 |
| 73 | *Bacillus altitudinis* | Cu1 | MPA, 37°C | KP699784 |
| 74 | *Escherichia coli* | E. coli | LB, 37°C |  |
